# Supplementary material for: scBSP: a fast and accurate tool for identifying spatially variable features from high-resolution spatial omics data
Source: Bioinformatics. 2025 Oct 1;41(10):btaf554. doi: 10.1093/bioinformatics/btaf554 (PMC12574330; doi:10.1093/bioinformatics/btaf554)
Supplement: btaf554_Supplementary_Data [file btaf554_supplementary_data.docx]

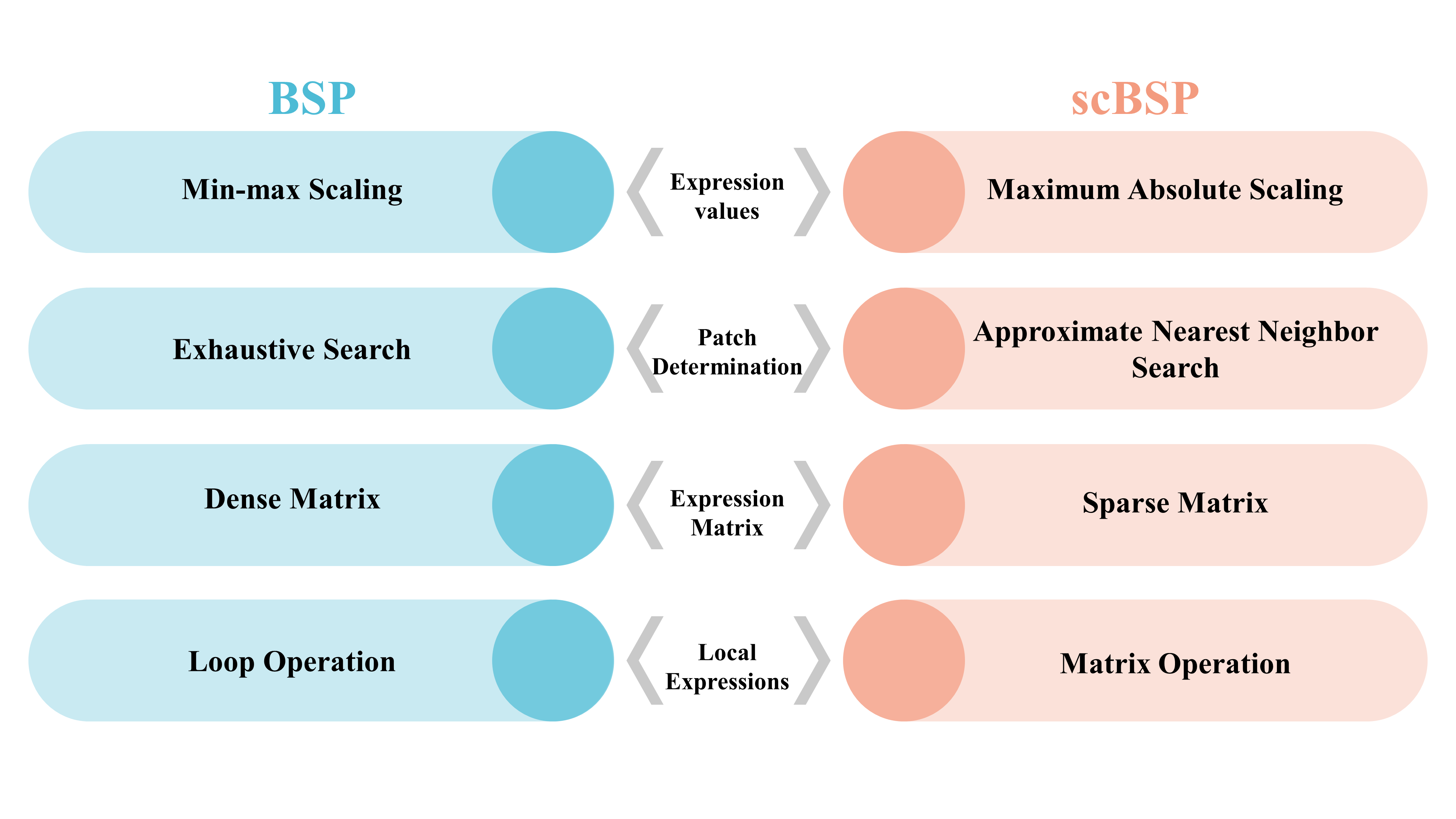


Supplementary Figure 1. Comparison between scBSP and BSP.


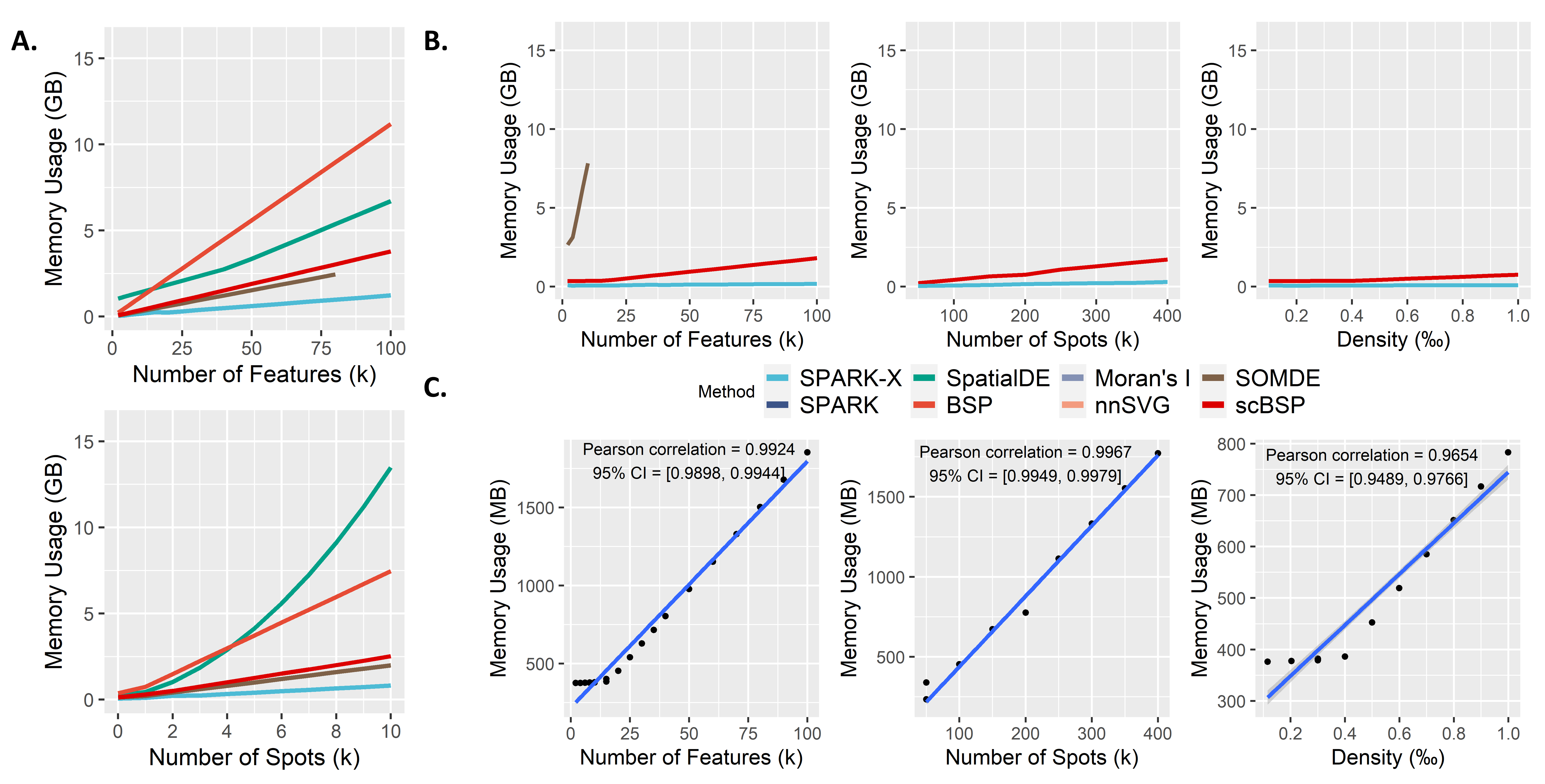


Supplementary Figure 2. A: Memory usage (y-axis) for analyzing spatial omics data comprising 20,000 features across 3,000 spots. B: Memory usage (y-axis) for analyzing high-resolution spatial omics data comprising 20,000 features across 100,000 spots, with a data density of 0.0005. This analysis varies one parameter while keeping the other two constants. C: Memory usage (y-axis) of scBSP on the high-resolution spatial omics data (run n = 10 times on a single processor core) with a varied number of features, spot count, and data density (x-axis).


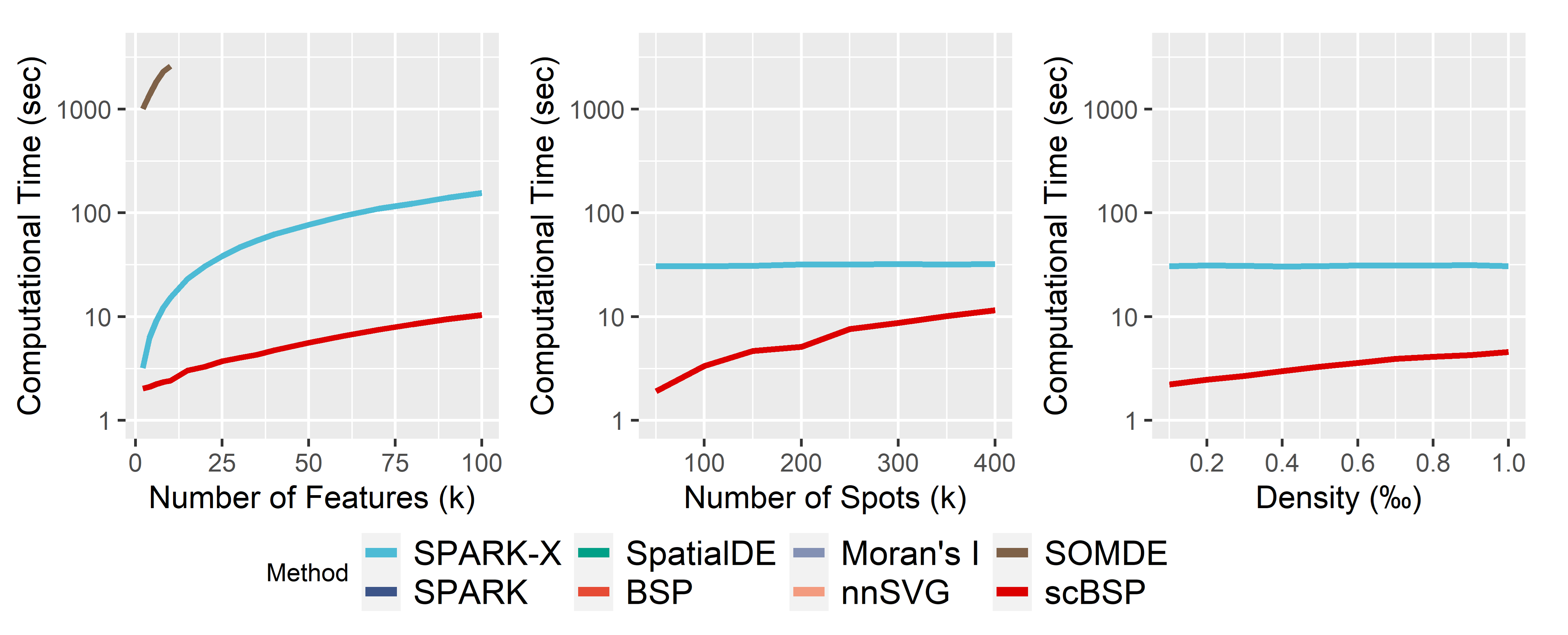


Supplementary Figure 3. Computational time (y-axis) for analyzing high-resolution spatial omics data comprising 20,000 genes across 100,000 spots, with a data density of 0.0005. This analysis varies one parameter while keeping the other two constants.


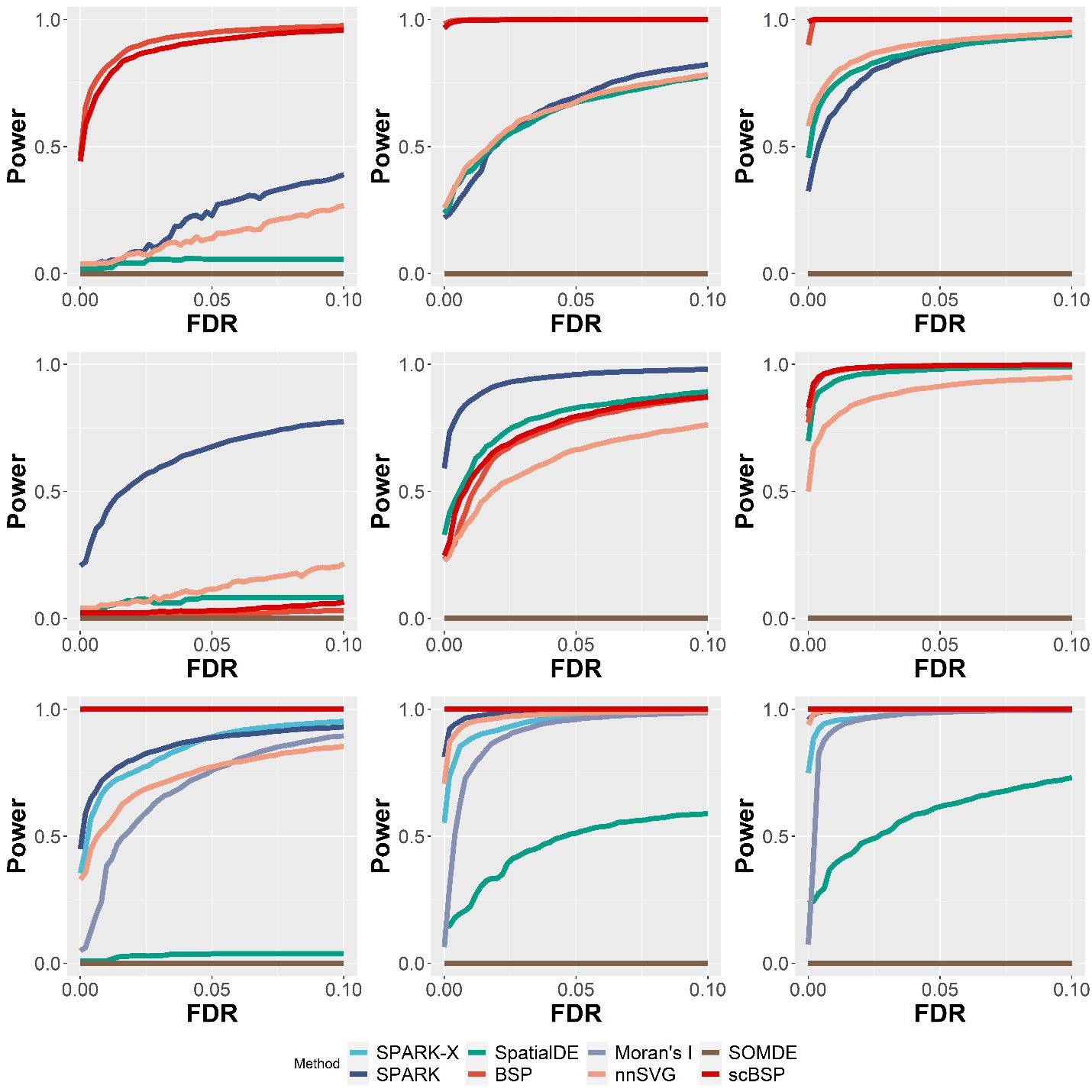


Supplementary Figure 4. Statistical power on 2D simulations with varied signal strengths. Signal strengths were measured as the fold changes in the averaged expressions between the pattern and non-pattern regions. Power curves were drawn using the averaged statistical power (y-axis) across ten replicates against the false discovery rates (x-axis) for the detected SVGs from each method. Results with weak (FC = 3), moderate (FC = 4), and high (FC = 5) signal strengths are shown in the left, middle, and right columns, while the upper, middle, and bottom rows represent the results from three spatial patterns in Figure 1D. All simulations were generated using a fixed moderate noise level.


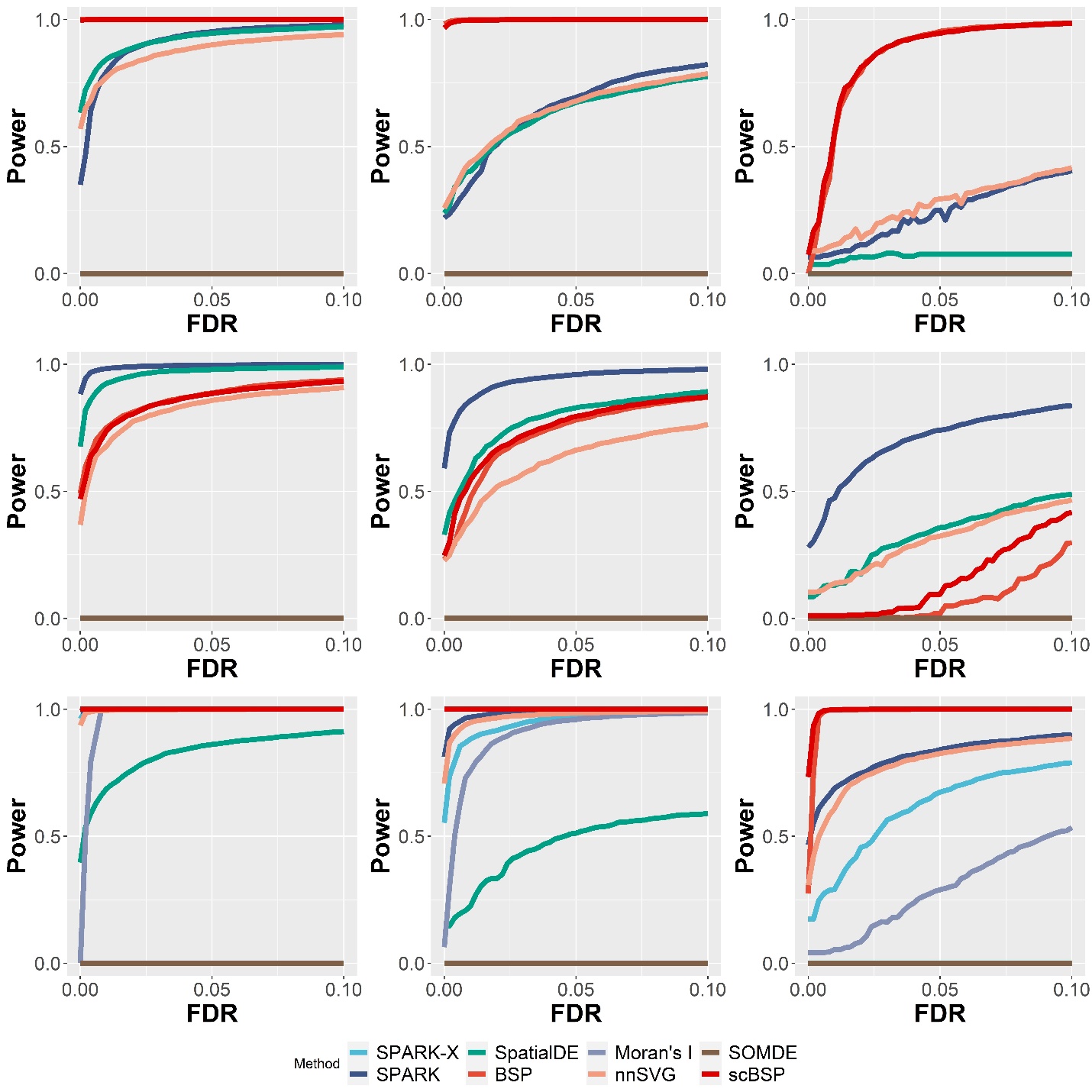


Supplementary Figure 5. Statistical power on 2D simulations with varied noise levels. Noise levels were defined as the dispersion parameters ($\tau_{2}$) in SPARK’s model. Power curves were drawn using the averaged statistical power (y-axis) across ten replicates against the false discovery rates (x-axis) for the detected SVGs from each method. Results with low ($\tau_{2}$=0.2), moderate ($\tau_{2}$=0.5), and high ($\tau_{2}$=0.8) noise levels are shown in the left, middle, and right columns, while the upper, middle, and bottom rows represent the results from three spatial patterns in Figure 1D. All simulations were generated using a fixed moderate signal strength.


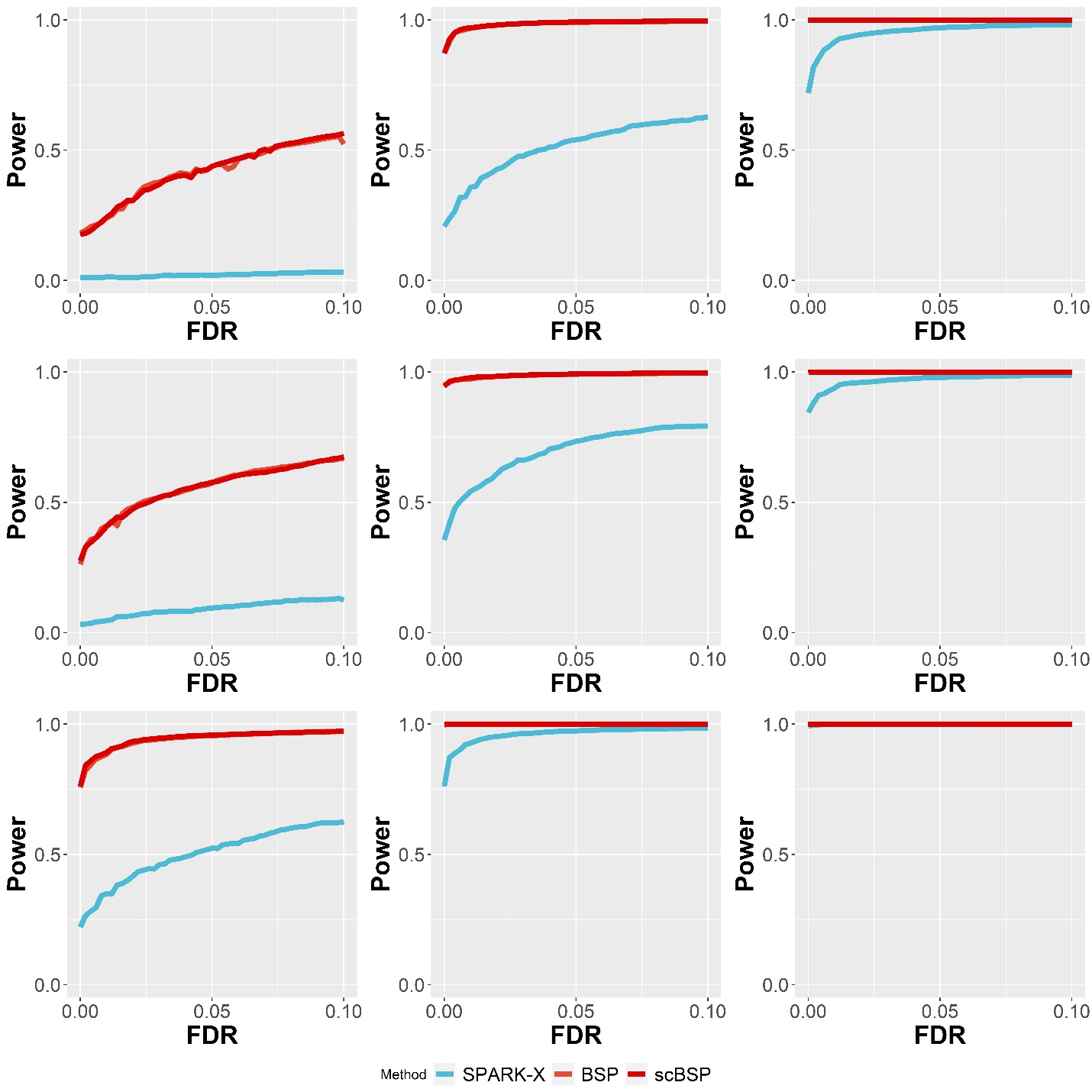


Supplementary Figure 6. Statistical power on 3D simulations of continuous patterns with varied pattern sizes. Pattern size was measured as the radius of the pattern as described in the Method section. Power curves were drawn using the averaged statistical power (y-axis) across ten replicates against the false discovery rates (x-axis) for the detected SVGs from each method. Results with small (radius=1.5), moderate (radius=2.0), and large (radius=2.5) pattern sizes are shown in the left, middle, and right columns, while the upper, middle, and bottom rows represent the results from three continuous spatial patterns in Figure 1E. All simulations were generated using a fixed moderate signal strength and noise level.


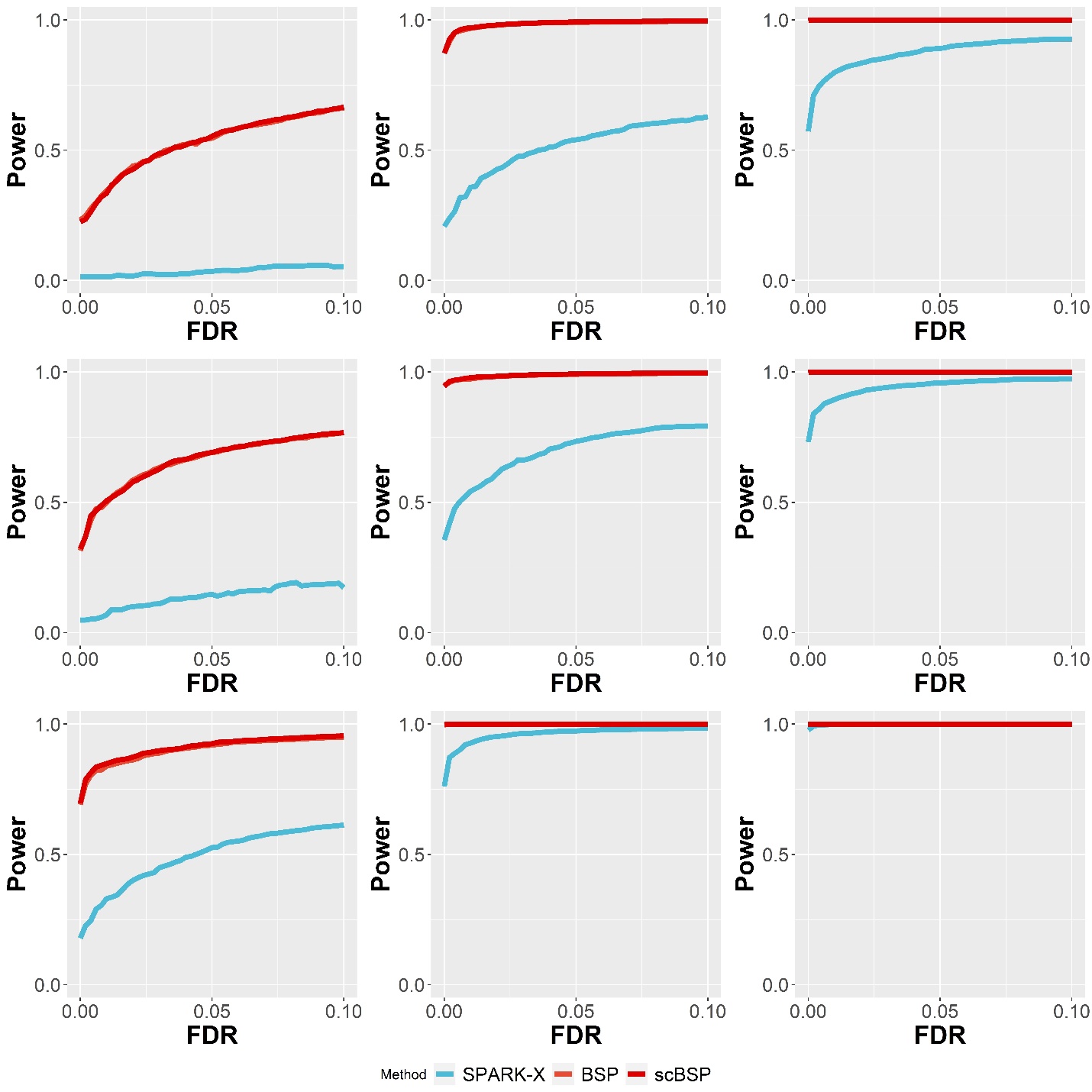


Supplementary Figure 7. Statistical power on 3D simulations of continuous patterns with varied signal strengths. Signal strengths were measured as the fold changes in the averaged expressions between the pattern and non-pattern regions. Power curves were drawn using the averaged statistical power (y-axis) across ten replicates against the false discovery rates (x-axis) for the detected SVGs from each method. Results with weak (FC = 2.0), moderate (FC = 2.5), and high (FC = 3.0) signal strengths are shown in the left, middle, and right columns, while the upper, middle, and bottom rows represent the results from three continuous spatial patterns in Figure 1E. All simulations were generated using a fixed moderate pattern size and noise level.


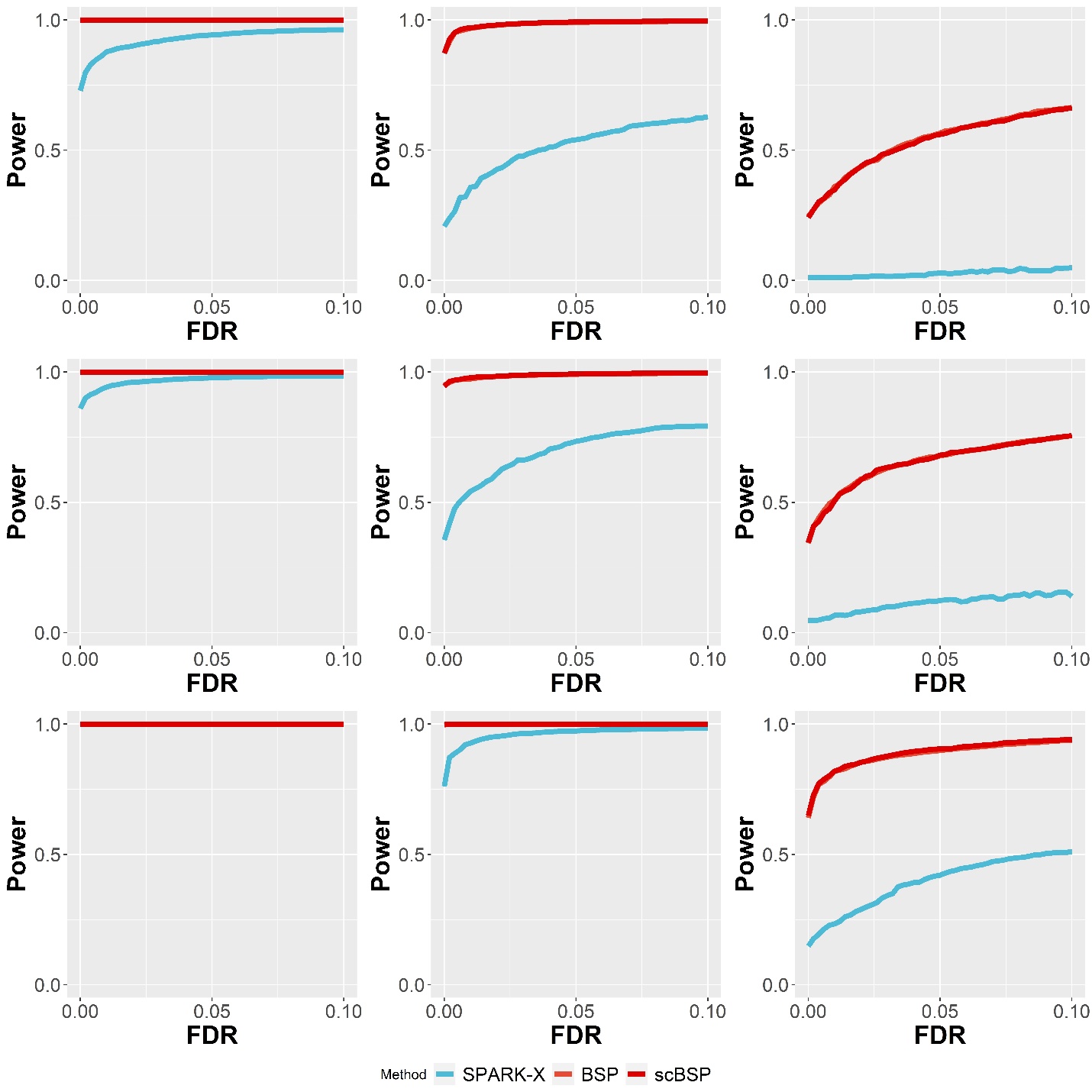


Supplementary Figure 8. Statistical power on 3D simulations of continuous patterns with varied noise levels. Noise levels ($\tau$) were measured as the proportions to the averaged standard deviation of simulated genes (detailed in the Method section). Power curves were drawn using the averaged statistical power (y-axis) across ten replicates against the false discovery rates (x-axis) for the detected SVGs from each method. Results with low ($\tau=0$), moderate ($\tau=1$), and high ($\tau=2$) noise levels are shown in the left, middle, and right columns, while the upper, middle, and bottom rows represent the results from three continuous spatial patterns in Figure 1E. All simulations were generated using a fixed moderate pattern size and signal strength.


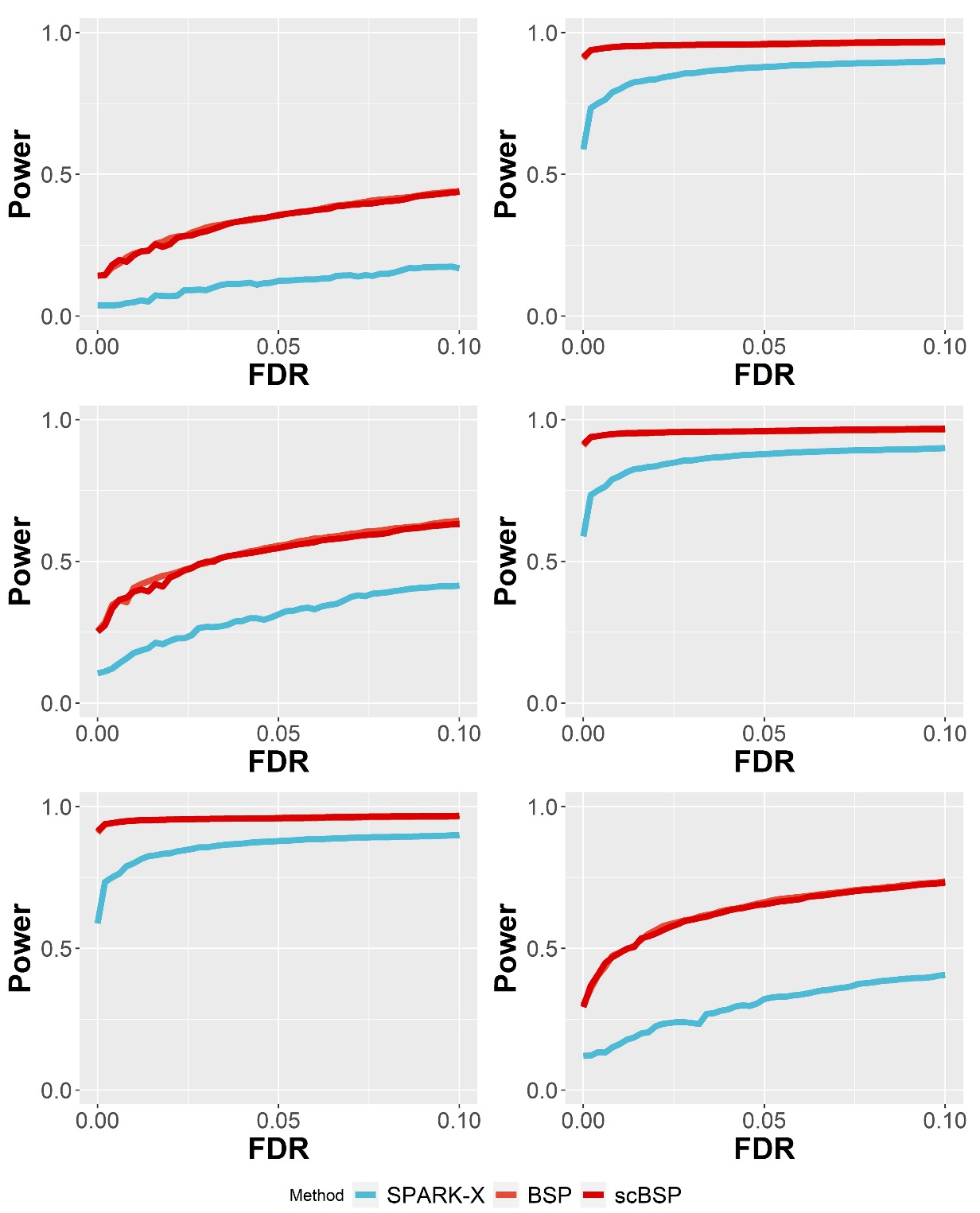


Supplementary Figure 9. Statistical power on 3D simulations of discrete patterns. Power curves were drawn using the averaged statistical power (y-axis) across ten replicates against the false discovery rates (x-axis) for the detected SVGs from each method. Results with varied pattern size (left: radius=1.5; right: radius=2.0), signal strengths (left: FC=2.0; right: FC=2.5) and noise levels (left: $\tau=2.0$; right: $\tau=3.0$) are shown in the upper, middle, and bottom rows as described in the Method section.


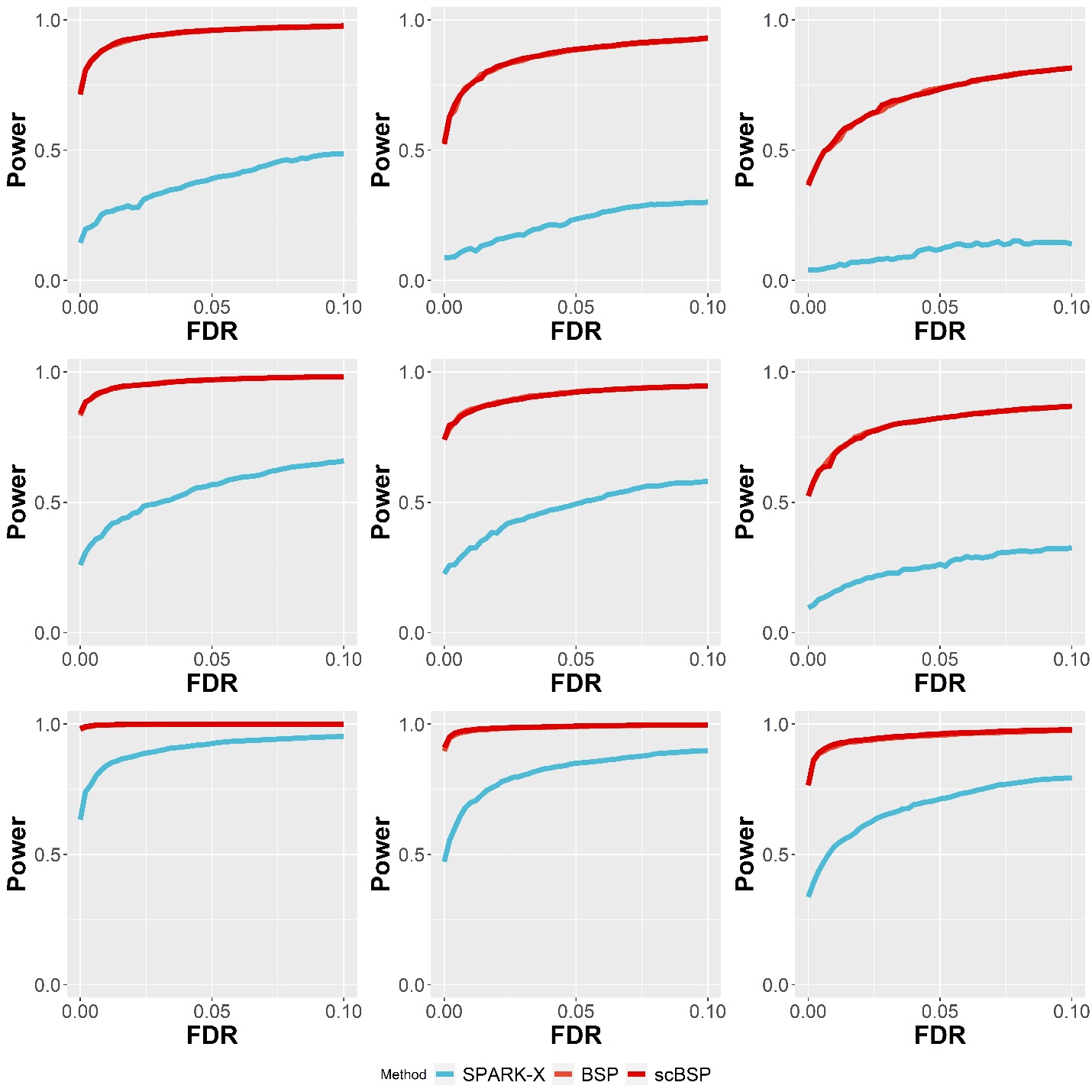


Supplementary Figure 10. Statistical power on 3D simulations of continuous patterns with varied dropout rates. Power curves were drawn using the averaged statistical power (y-axis) across ten replicates against the false discovery rates (x-axis) for the detected SVGs from each method. Results with low (10%), moderate (20%), and high (30%) dropout rates are shown in the left, middle, and right columns, while the upper, middle, and bottom rows represent the results from three continuous spatial patterns in Figure 1E. All simulations were generated using a fixed moderate pattern size and signal strength.


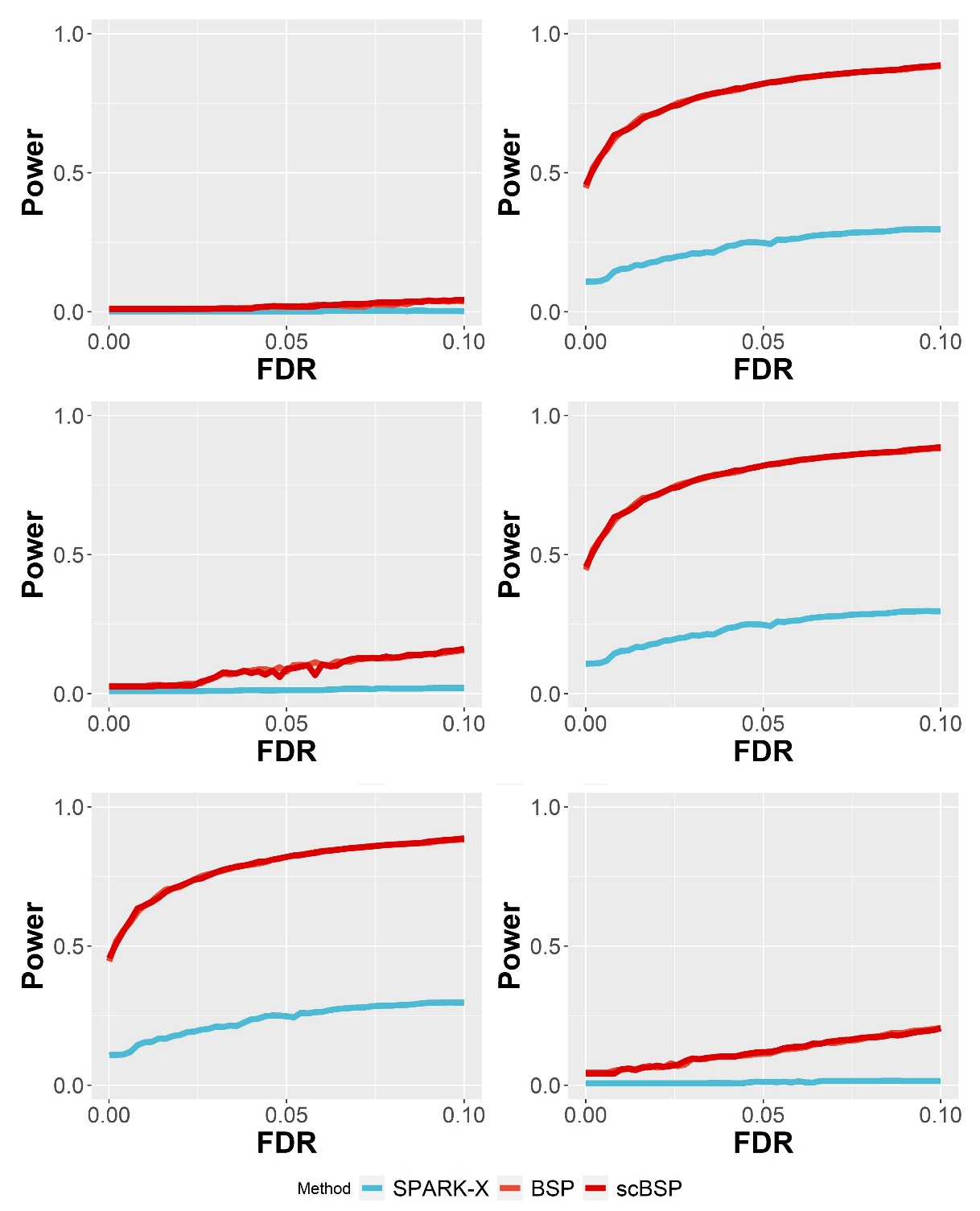


Supplementary Figure 11. Statistical power on 3D simulations of with inconsistent inter-plane and within-plane spatial resolution. Power curves were drawn using the averaged statistical power (y-axis) across ten replicates against the false discovery rates (x-axis) for the detected SVGs from each method. Results with varied pattern size (left: radius=1.5; right: radius=2.0), signal strengths (left: FC=2.0; right: FC=2.5) and noise levels (left: $\tau=1.0$; right: $\tau=2.0$) are shown in the upper, middle, and bottom rows as described in the Method section.


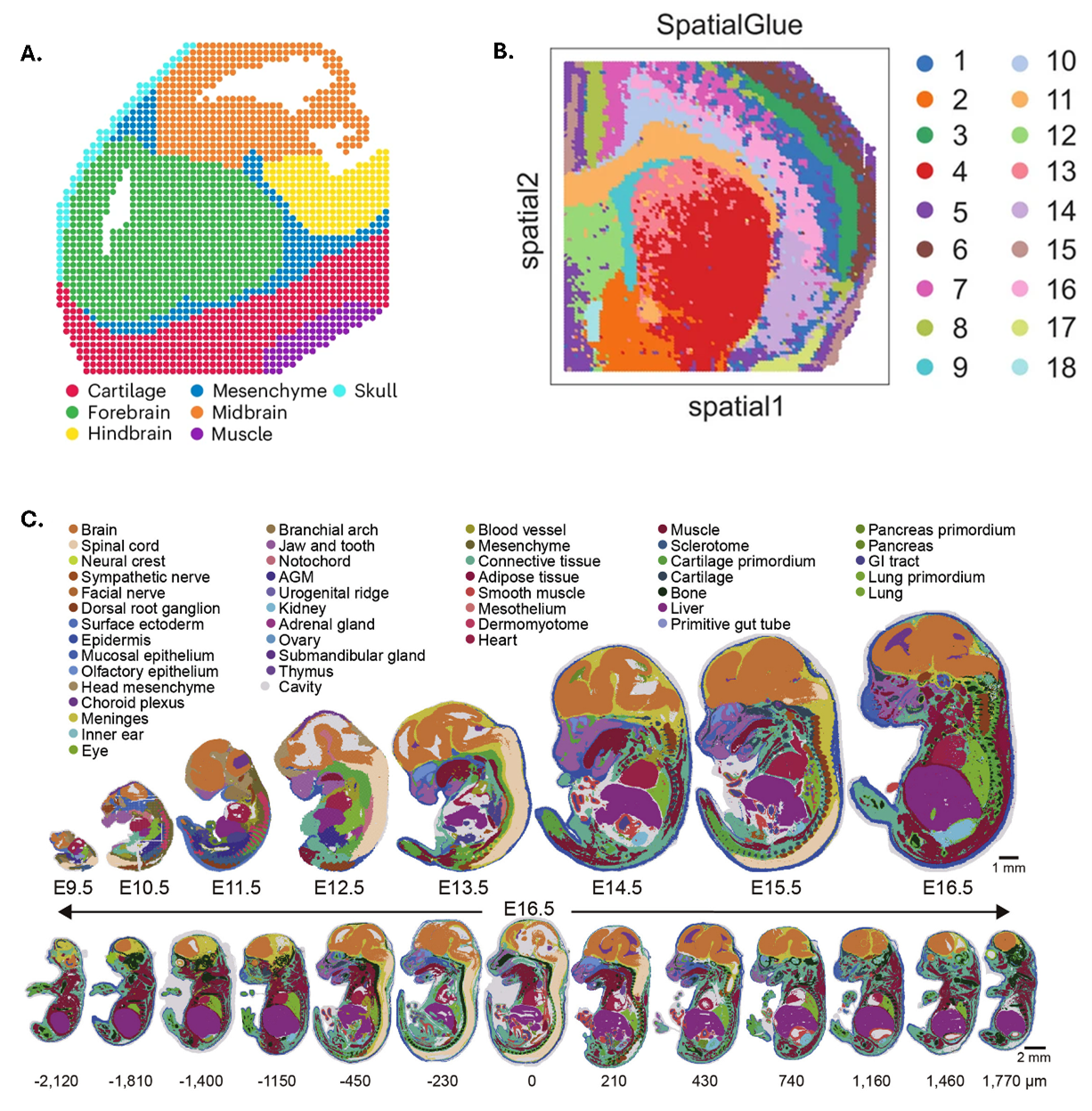


Supplementary Figure 12. Tissue architecture of spatial datasets used in analysis. **A:** Annotation for mouse embryonic (E15.5) brain tissues in the MISAR-seq dataset by Tian et al. (2024). **B:** Clusters identified by *SpatialGlue* for Long et al. (2024) in the mouse brain spatial-ATAC-RNA-seq P22 sample. **C:** Original spatial atlas’s annotation of mouse organogenesis from Chen et al. (2022). Bins are colored by their annotation.


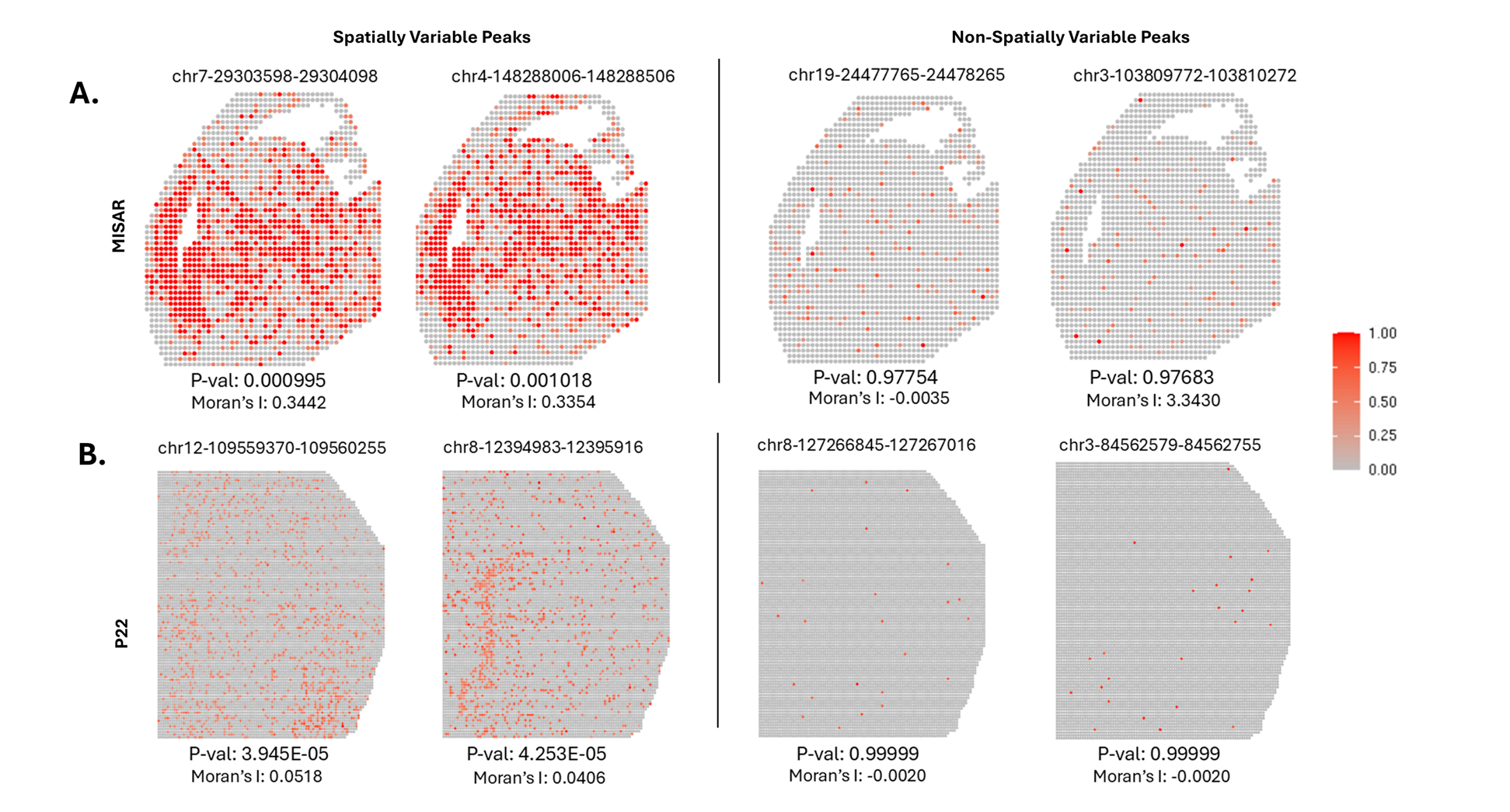


Supplementary Figure 13. Additional top significant and insignificant spatially variable peaks identified by scBSP. A: Additional top significant and insignificant spatially variable peaks on MISAR mouse brain dataset. B: Additional top significant and insignificant spatially variable peaks on the P22 mouse brain dataset.


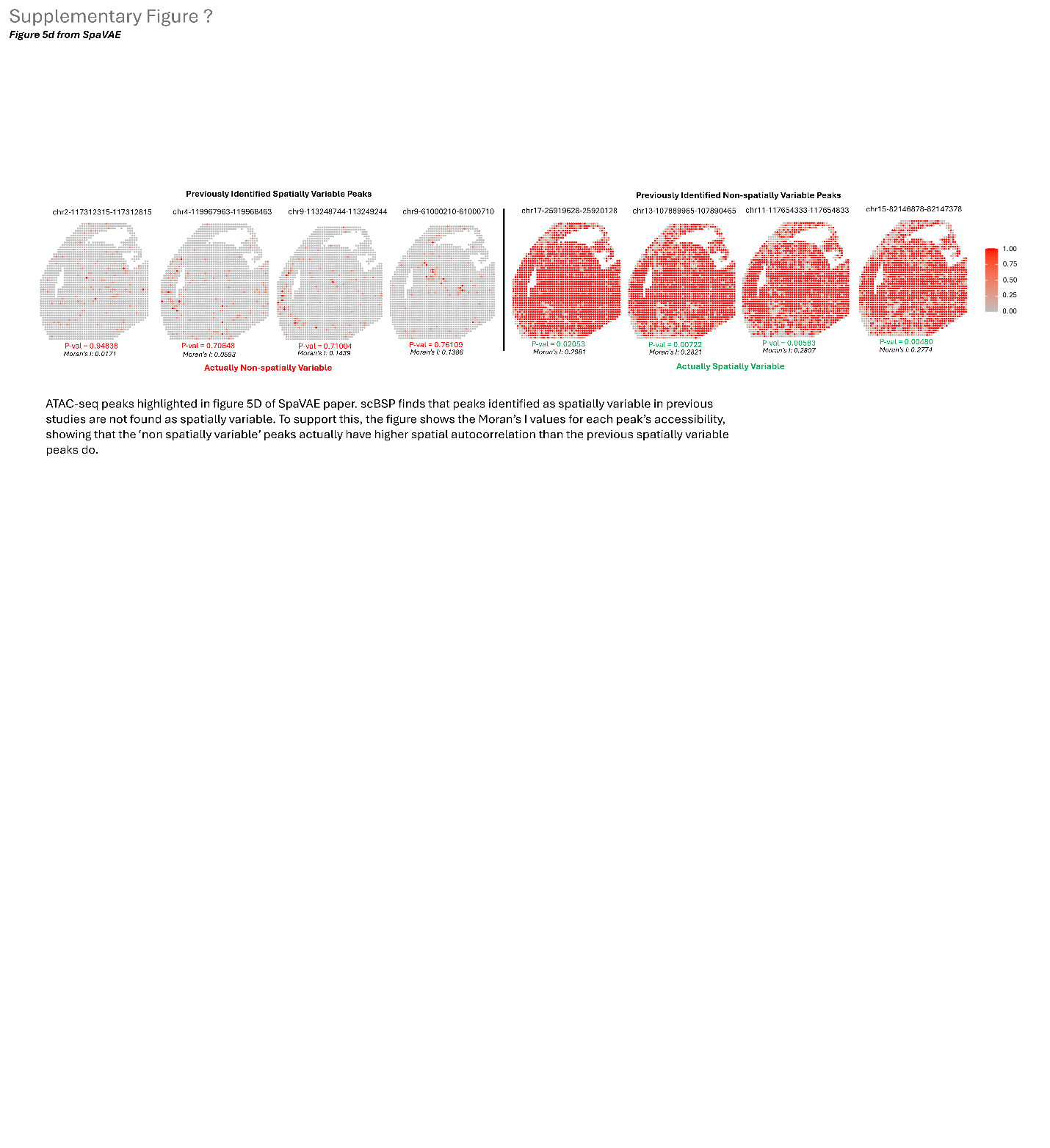


Supplementary Figure 14. Spatially variable ATAC-seq peaks highlighted in previous studies, which were not identified by scBSP (left), and ATAC-seq peaks identified by scBSP, which were not identified in previous work (right). The figure shows the Moran’s I values for each peak’s accessibility, showing that previous ‘non spatially variable’ peaks actually have higher spatial autocorrelation than the previous spatially variable peaks.


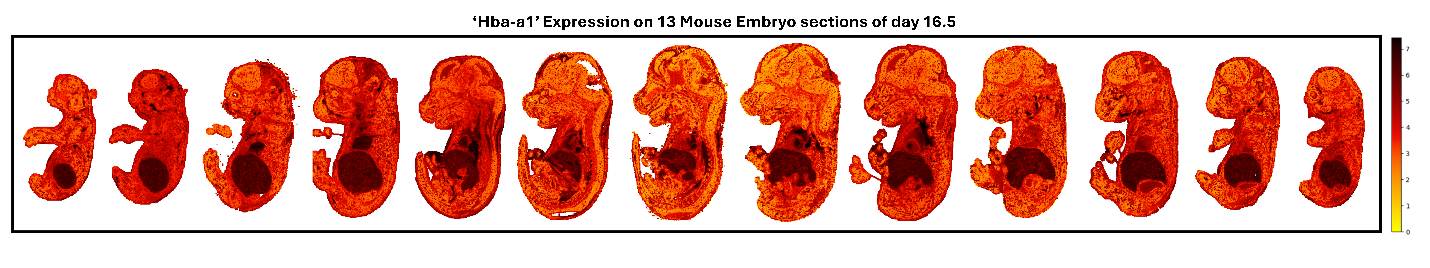


Supplementary Figure 15. Expression of Gene Hba-a1 on 13 consecutive sections of a 3D mouse embryo on day 16.5 with Stereo-seq. This identified SVF shows gene expression in the fetal liver and heart of the embryo.


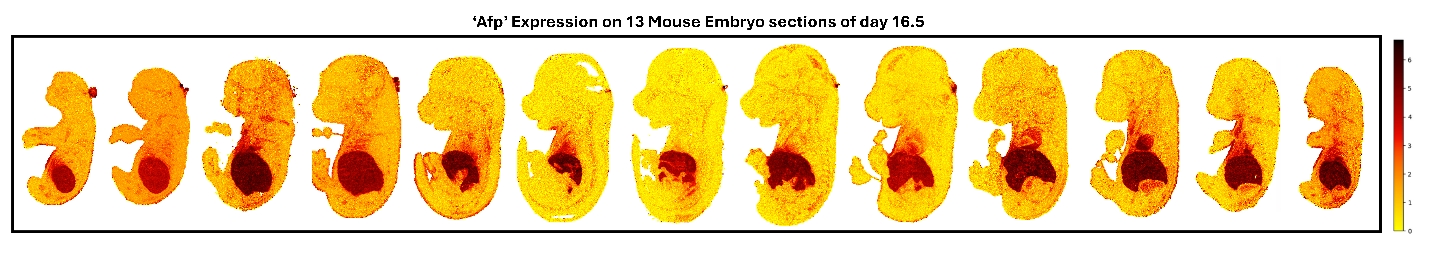


Supplementary Figure 16. Expression of Fetal Liver marker gene Afp on 13 consecutive sections of a mouse embryo on day 16.5 with Stereo-seq.


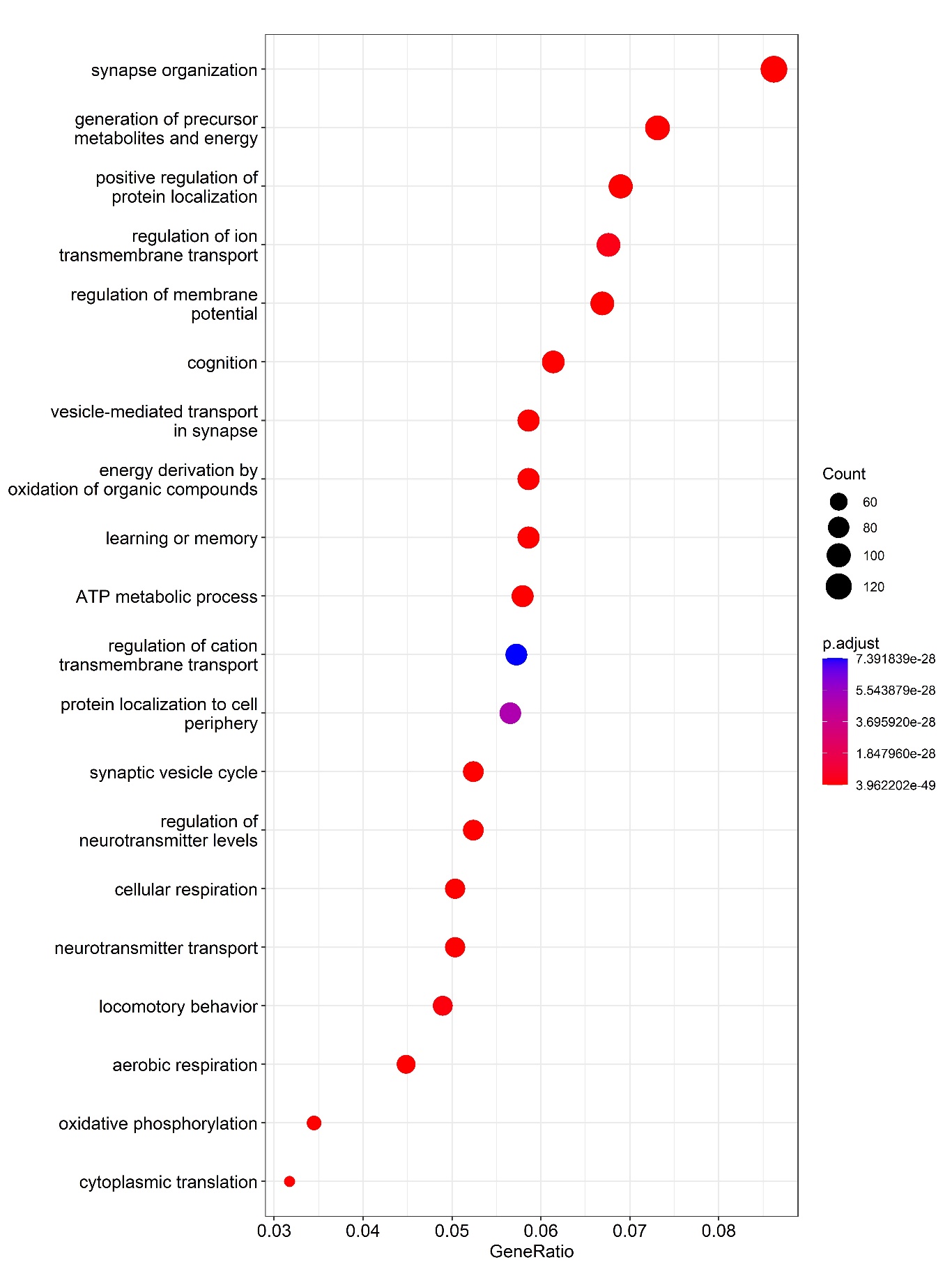


Supplementary Figure 17. Enriched gene ontology terms on 10x Visium mouse brain anterior 1 data.


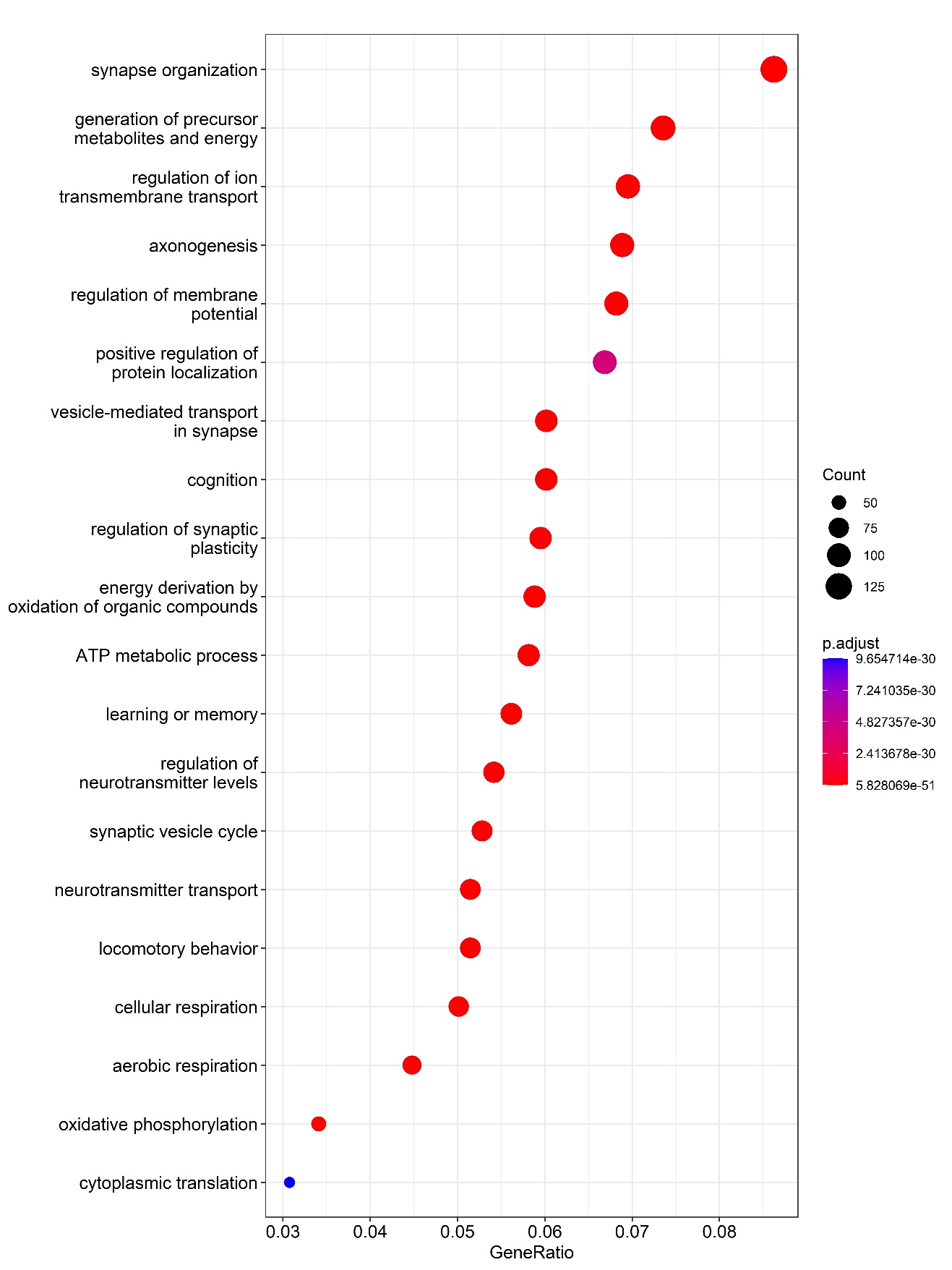


Supplementary Figure 18. Enriched gene ontology terms on 10x Visium mouse brain anterior 2 data.


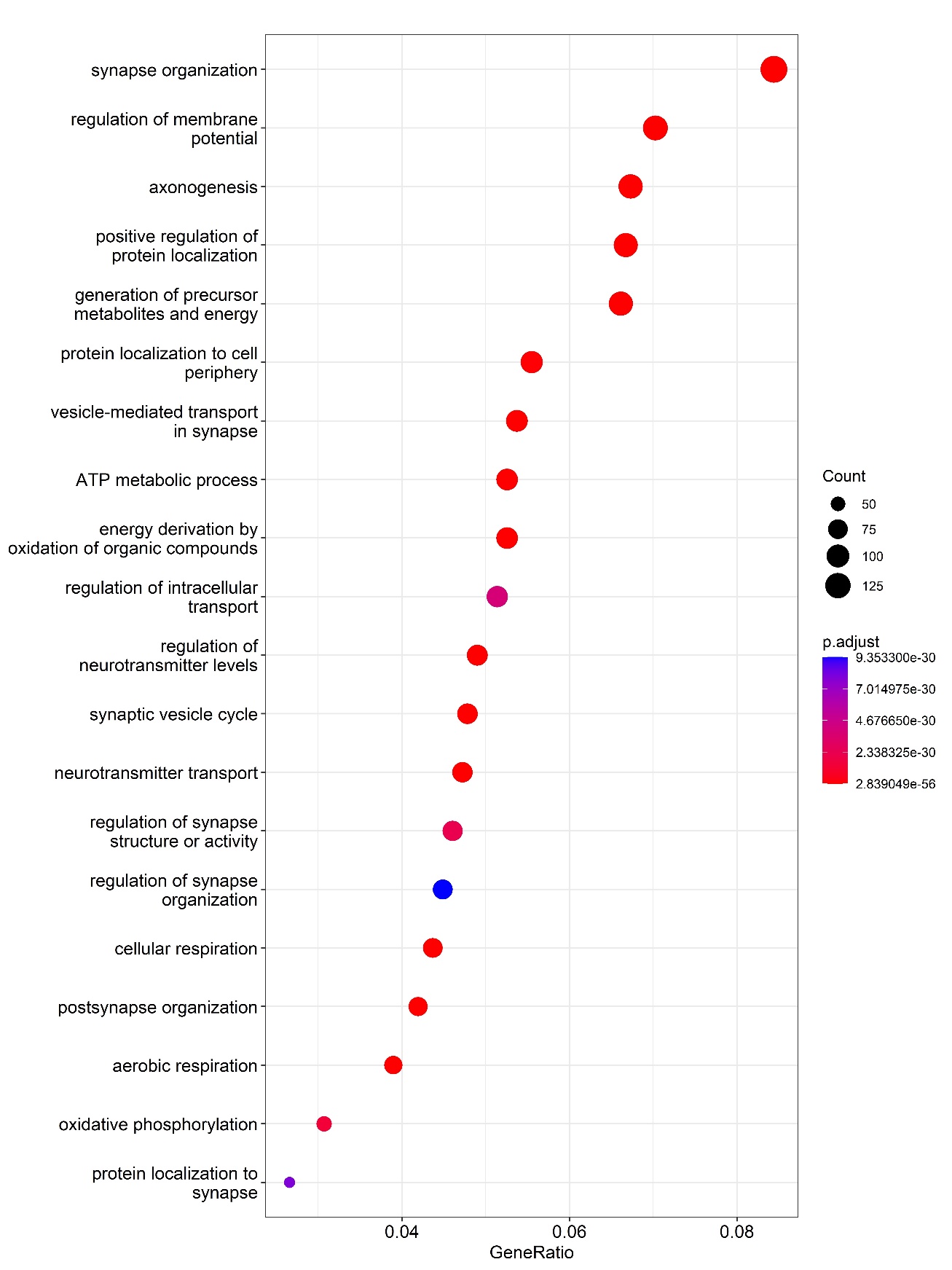


Supplementary Figure 19. Enriched gene ontology terms on 10x Visium mouse brain Posterior 1 data.


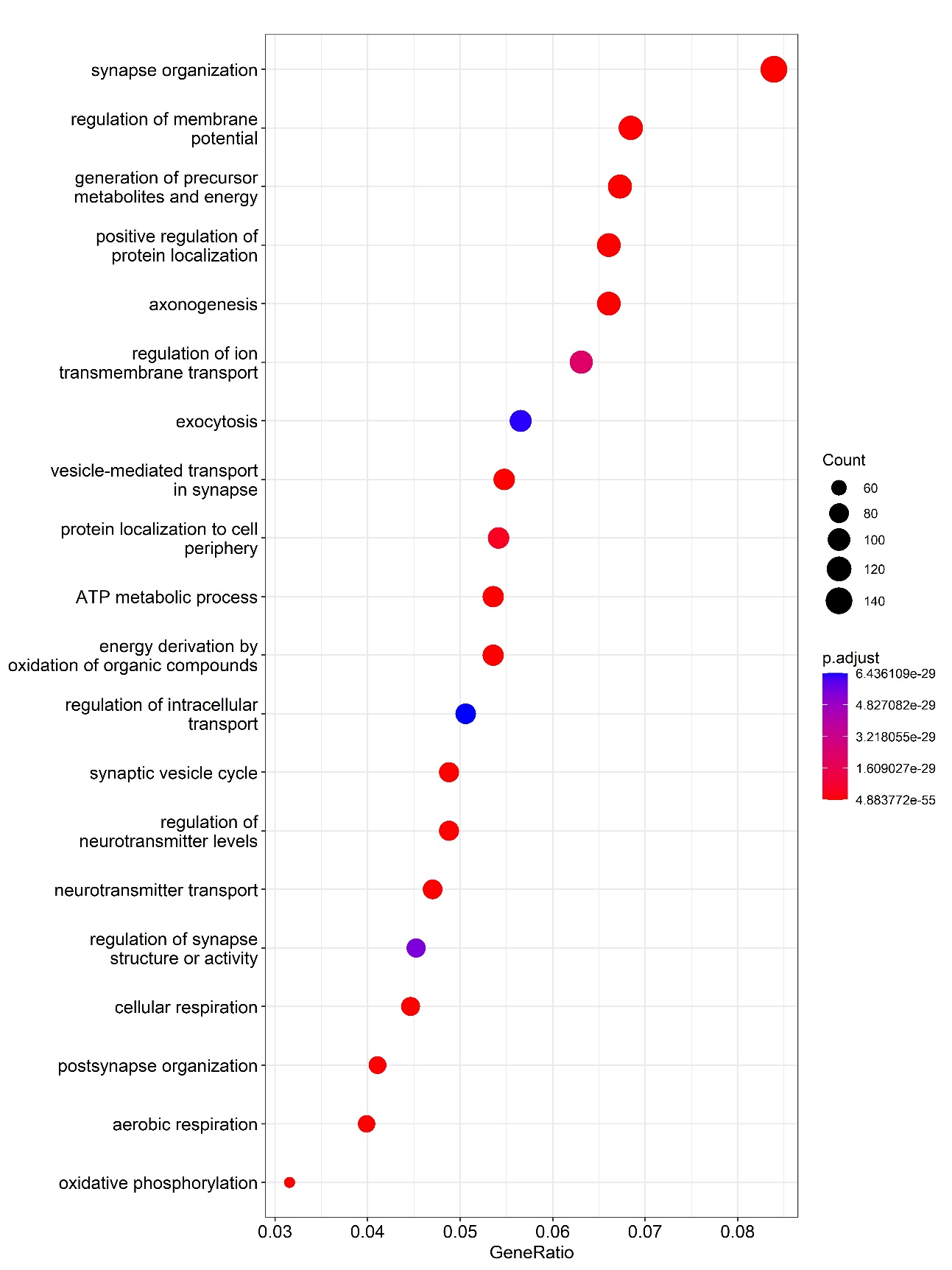


Supplementary Figure 20. Enriched gene ontology terms on 10x Visium mouse brain Posterior 2 data.


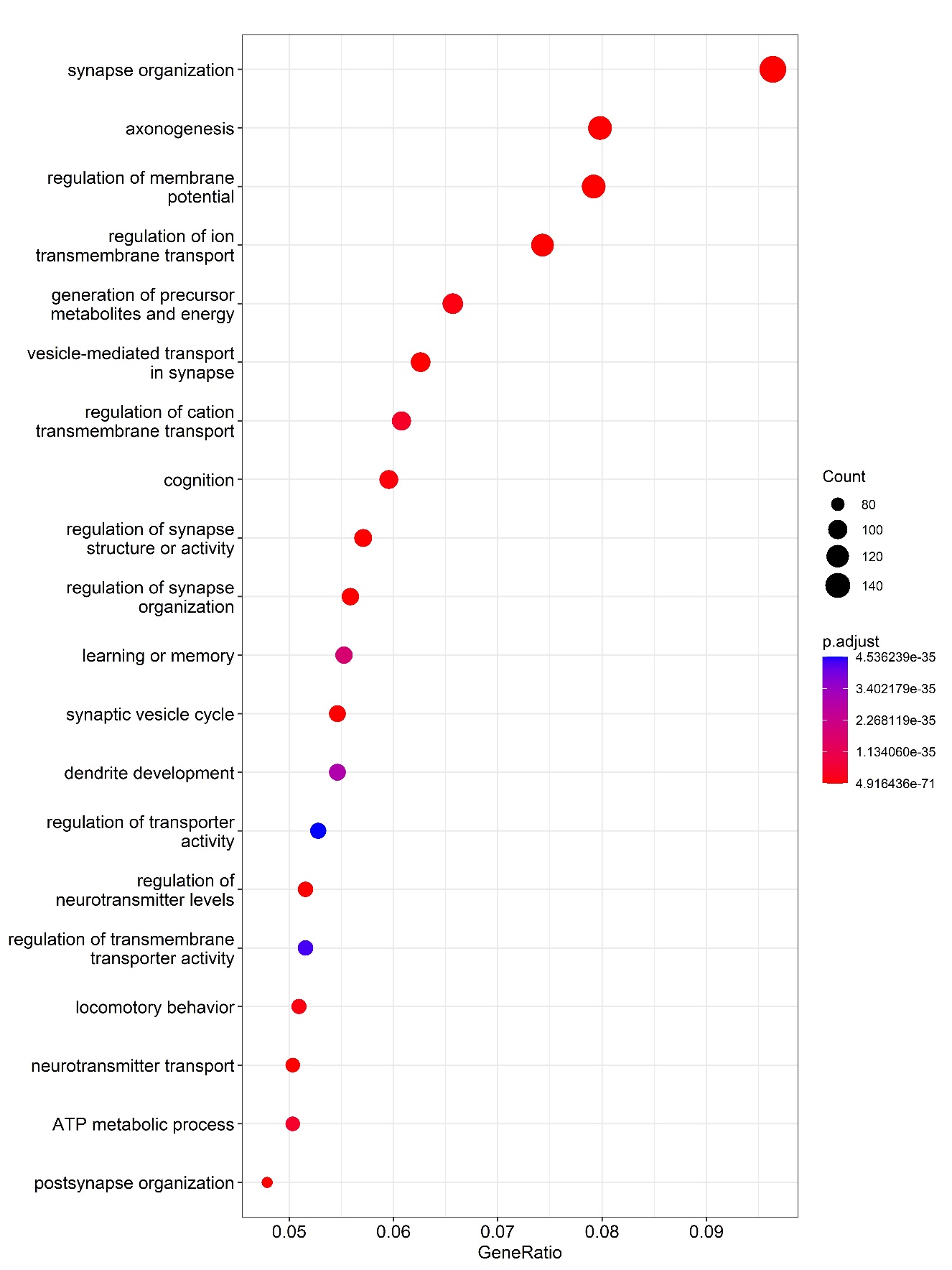


Supplementary Figure 21. Enriched gene ontology terms on Stereo-seq mouse brain data.


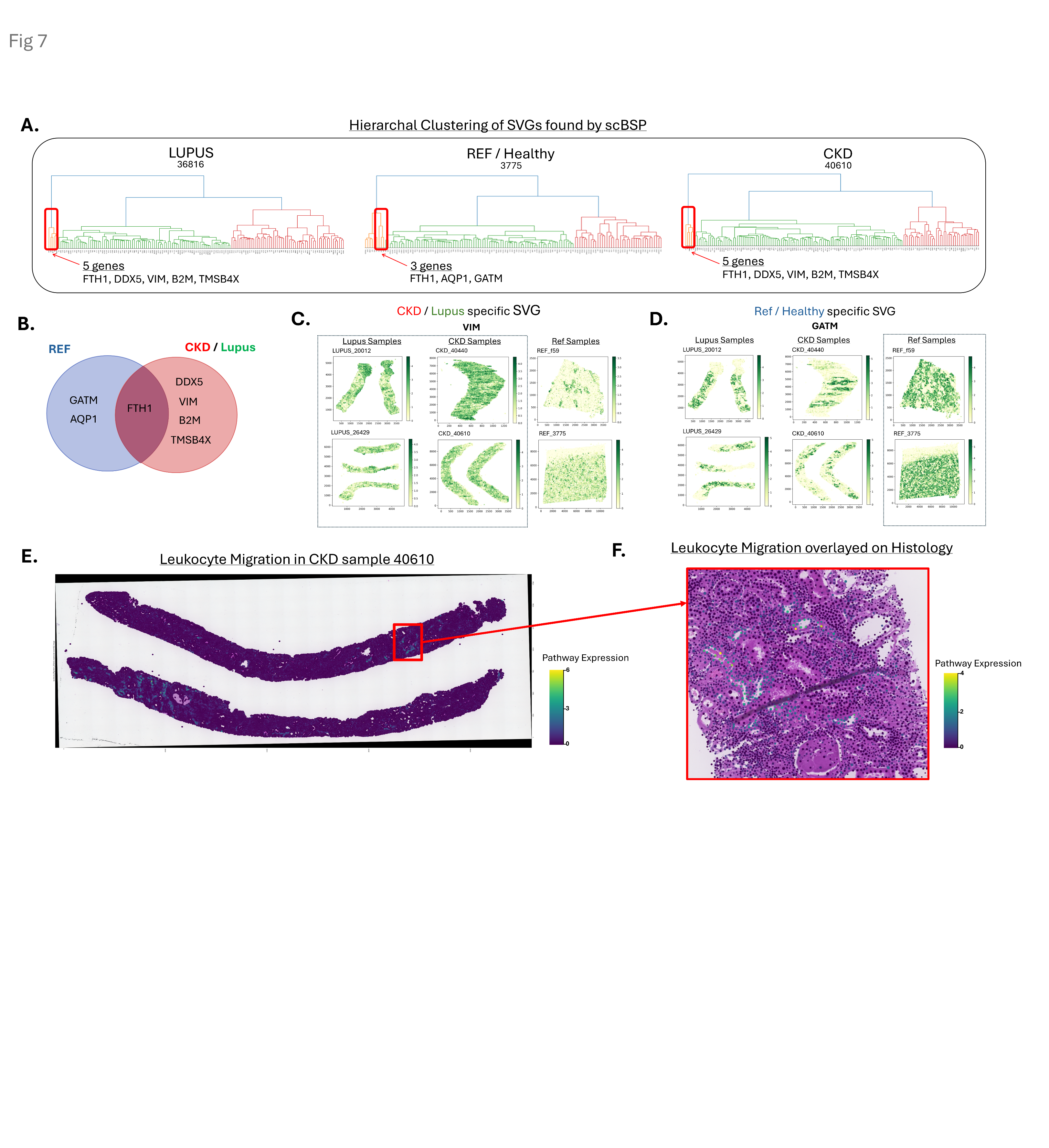


Supplementary Figure 22. Gene expression of the 10x Xenium kidney samples. This group plots the gene expression of GATM, a reference-specific SVG. The difference in expression is observed in the CKD/Lupus vs. reference samples.


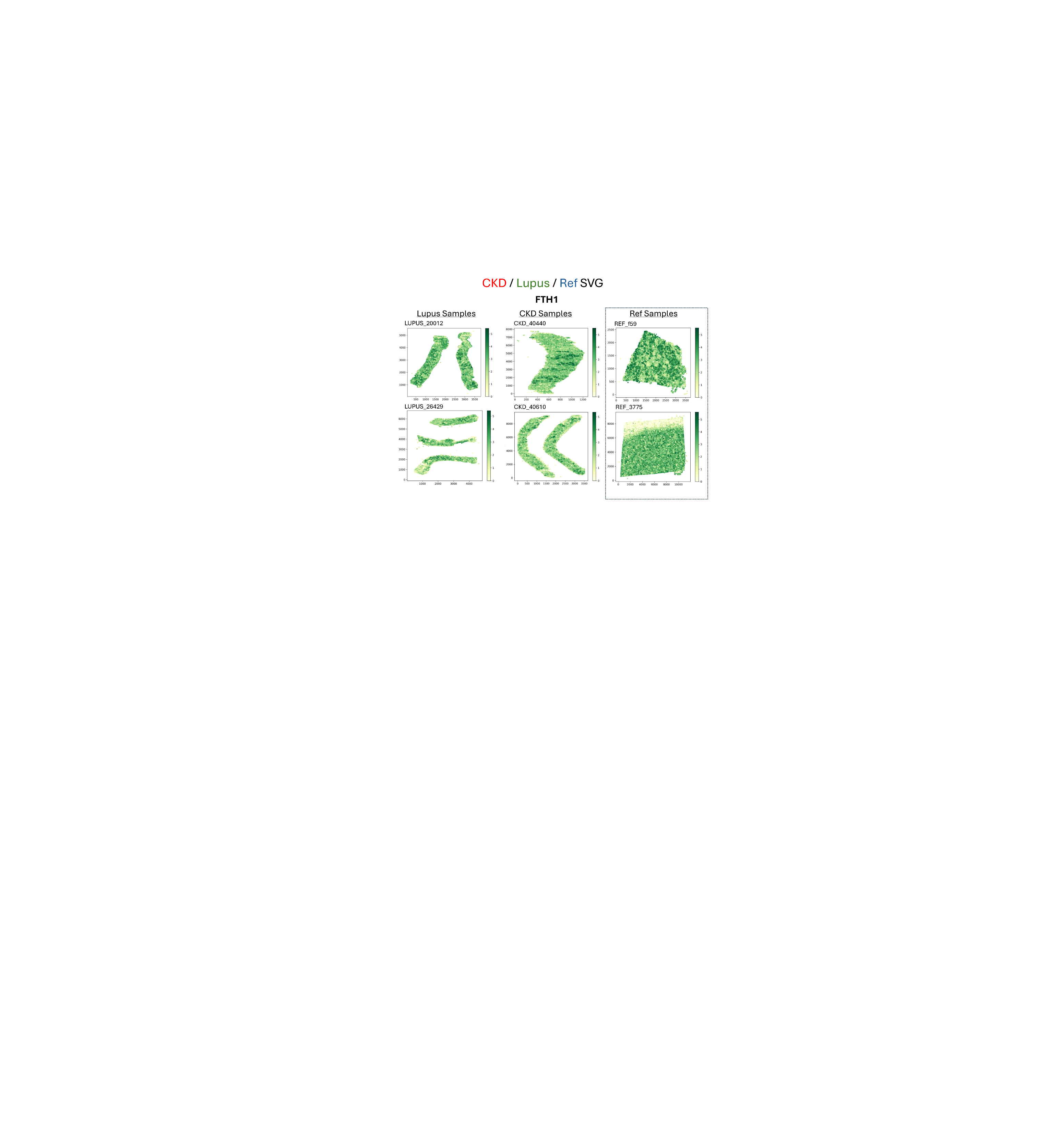


Supplementary Figure 23. Gene expression of the 10x Xenium kidney samples. This group plots the gene expression of FTH1, an SVG found for all three conditions analyzed.


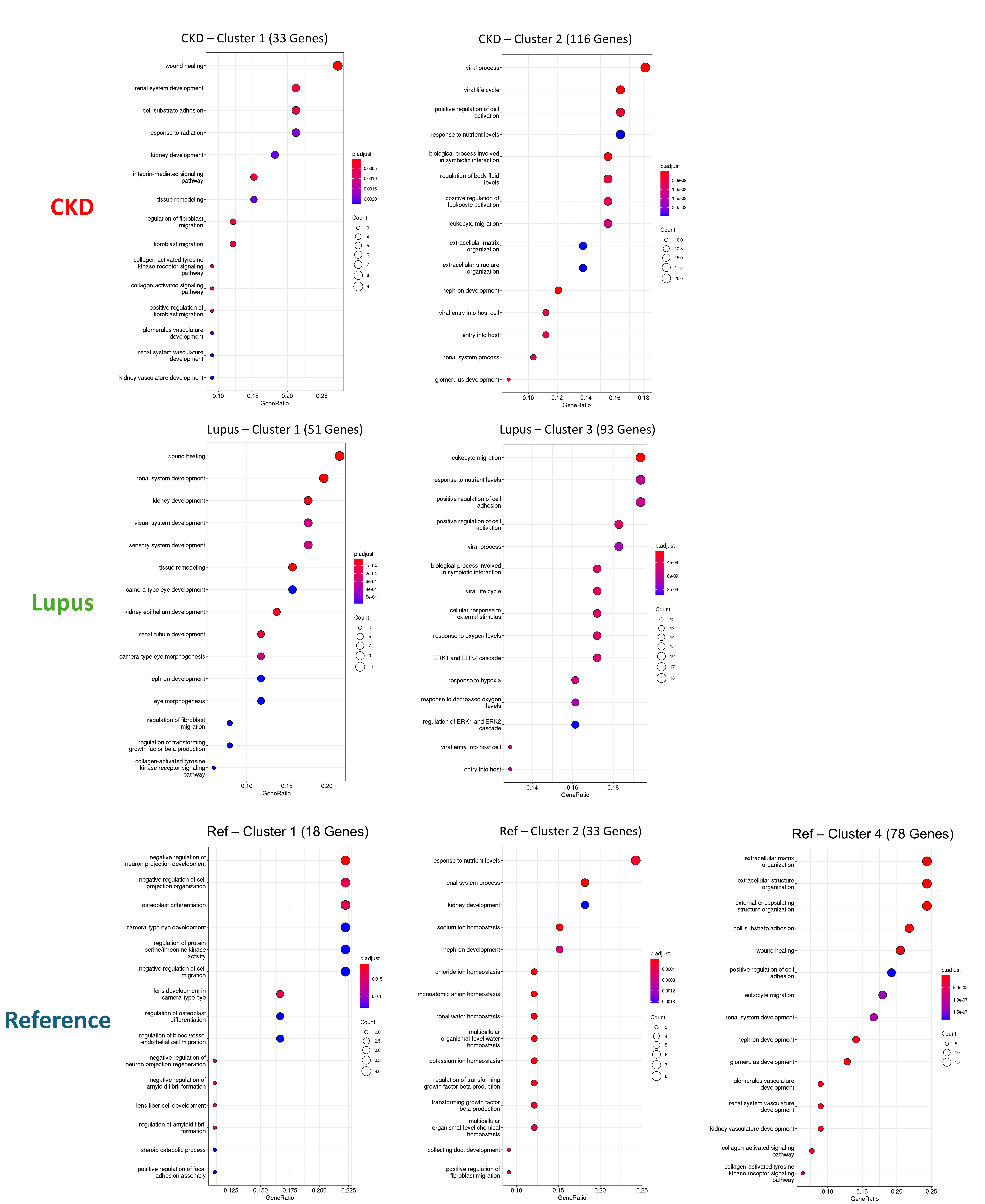


Supplementary Figure 24. Gene Ontology terms comparison between different condition clusters, with clusters of < 10 genes omitted.


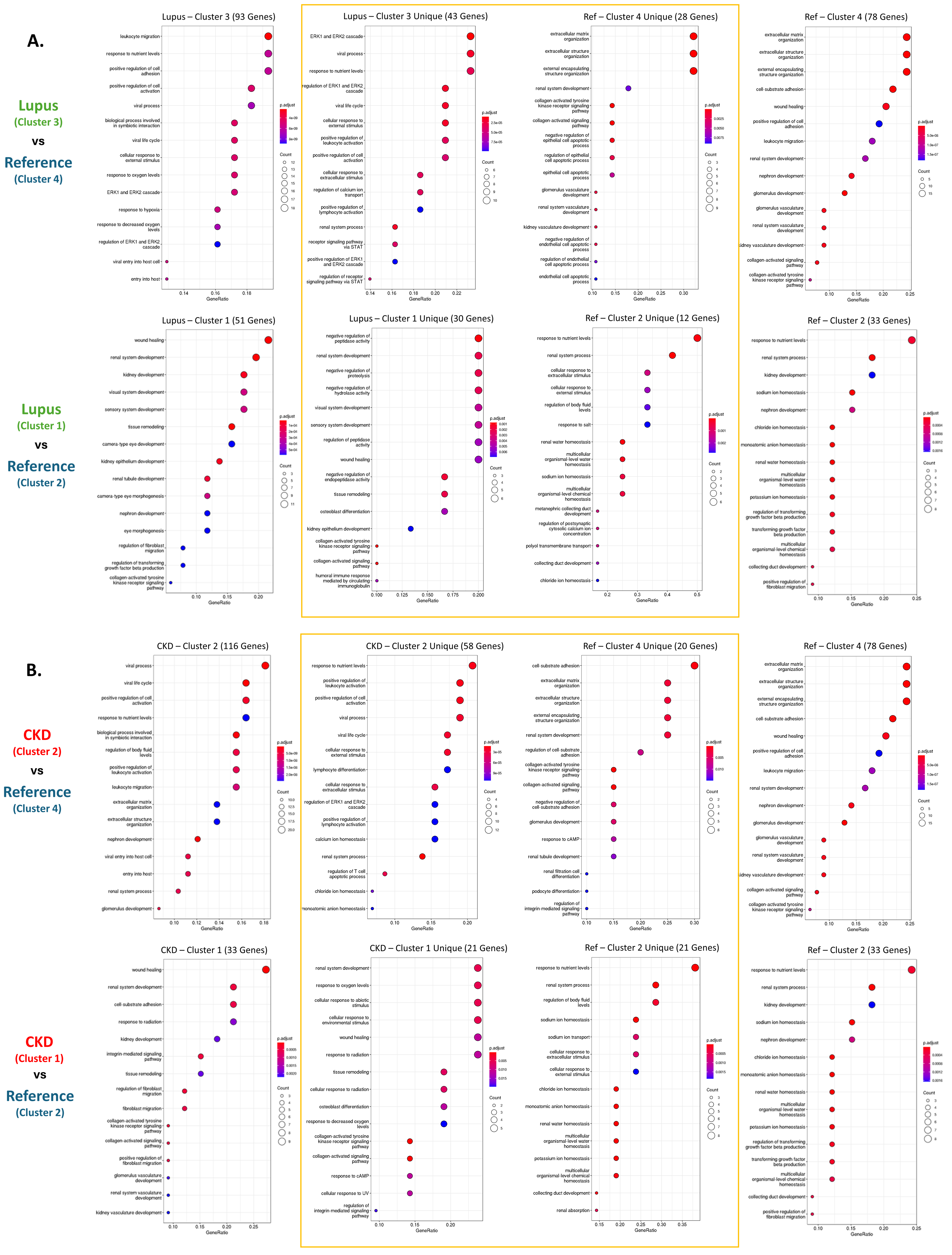


Supplementary Figure 25. Gene Ontology terms comparison between full clusters and unique genes within the cluster. **A.** Lupus vs. Reference condition comparisons **B.** CKD vs. Reference comparisons.


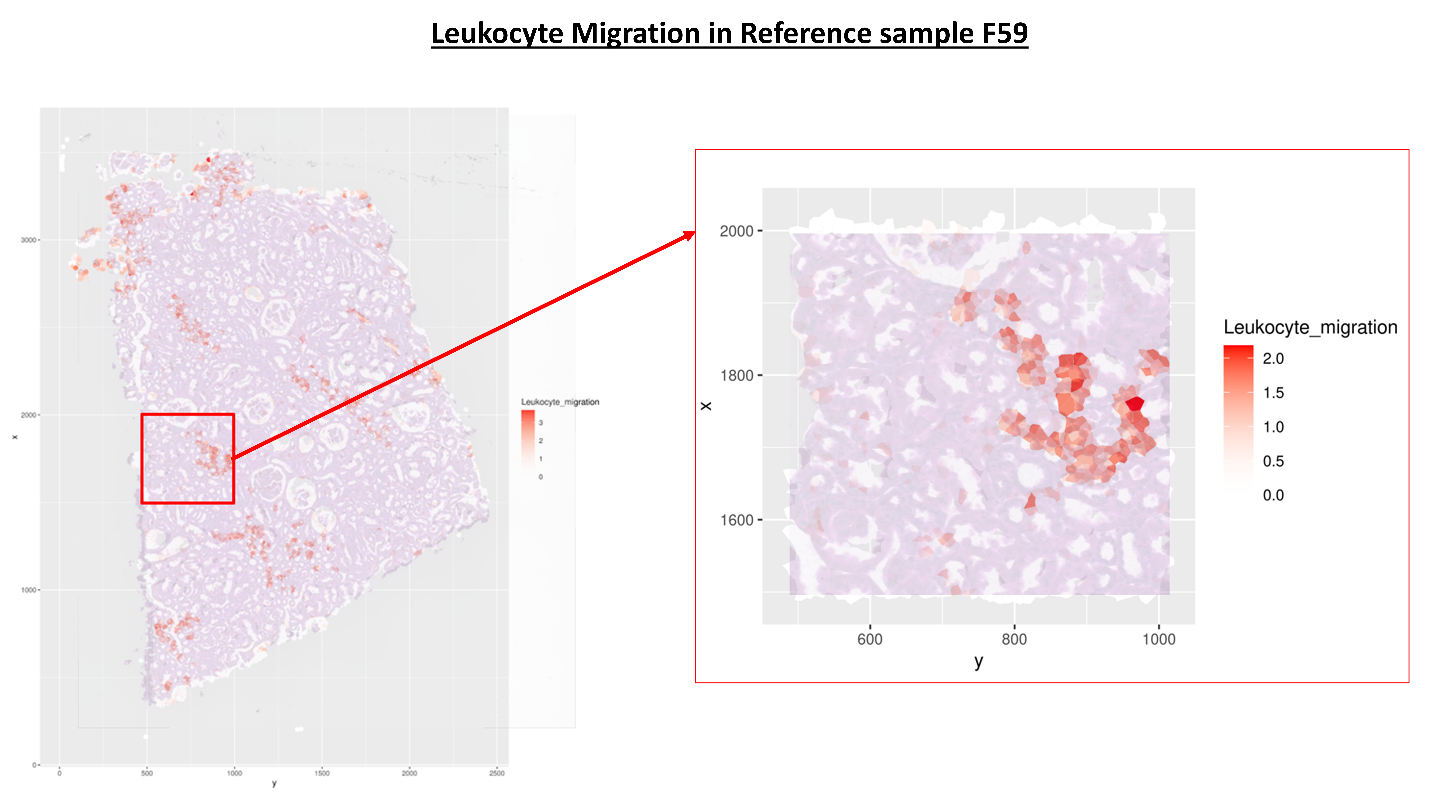


Supplementary Figure 26. Sample F59 – Reference, H&E image overlapped with pathway expression of leukocyte migration, showing both the full sample and a zoomed-in area of high pathway expression.


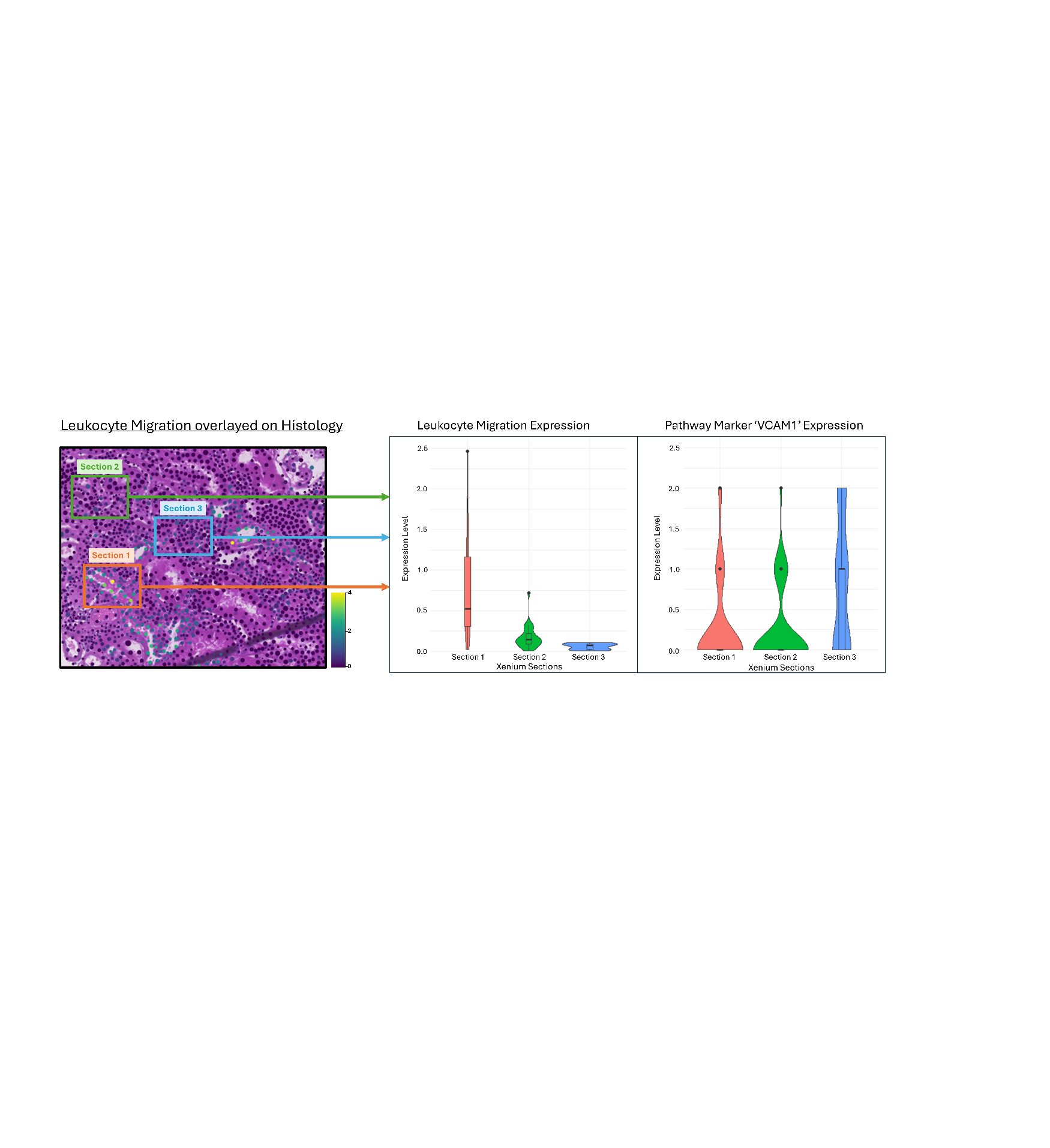


Supplementary Figure 27. Sample 40610 – CKD, identified sections of higher expression with surrounding areas of lower pathway expression. Violin expression plots show increased pathway expression in Section 1 compared to Sections 2 and 3, while gene expression of the marker gene VCAM1 shows increased expression in Section 3.


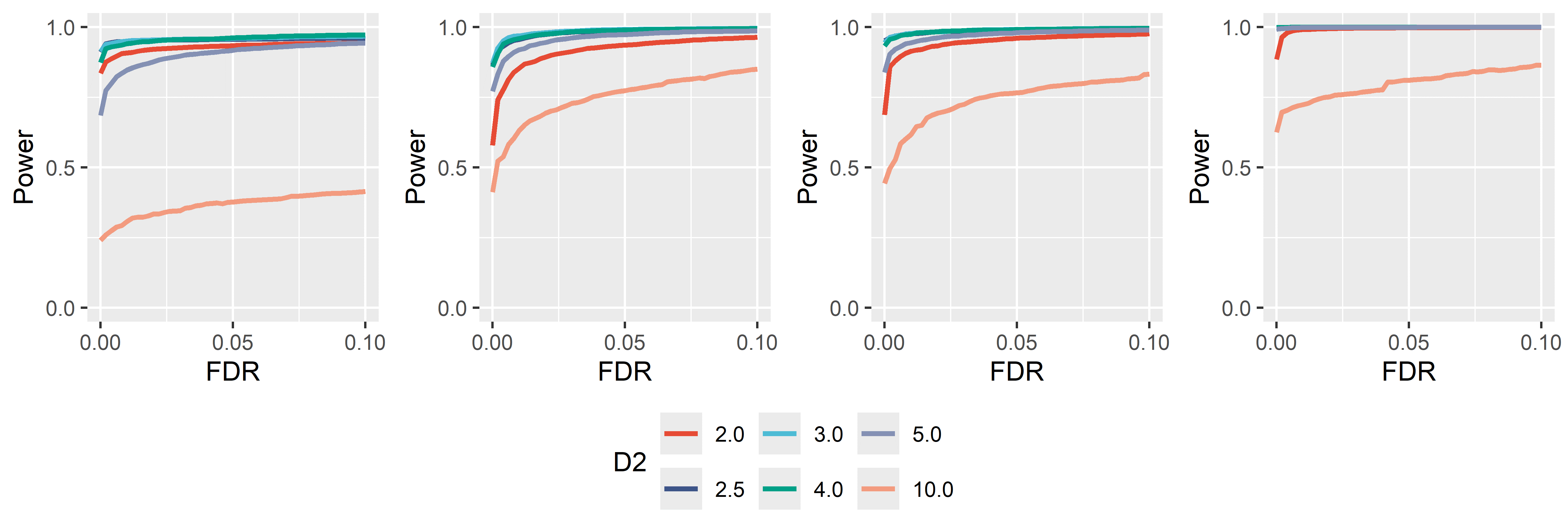
Supplementary Figure 28. Power comparison across different gradient scales. Power was evaluated by varying the size of the large patch while keeping the small patch fixed at one unit. Simulations were based on the 3D patterns with moderate pattern size, signal strength, and noise level, as illustrated in Figure 1E, with Isolated Cell Nodules patterns, Curved Cell Strand, Tissue Layer, and Irregular Cell Aggregate (left to right).
